# The Evolution and Impact of the Nasal Positive Pressure Oxygenation Device (SuperNO2VA™) in Modern Anesthesia

**DOI:** 10.7759/cureus.87221

**Published:** 2025-07-03

**Authors:** Vladislav P Zhitny, Brett Dixon, Ryan Jannoud, Joelle Sills, Ariana R Carillo, Michael C Wajda, Aaron N Primm, Brian Mendelson

**Affiliations:** 1 Department of Anesthesiology, Perioperative Care, and Pain Medicine, New York University Langone Health, New York, USA; 2 School of Medicine, Kirk Kerkorian School of Medicine, University of Nevada, Las Vegas, Las Vegas, USA; 3 School of Medicine, Duke University School of Medicine, Durham, USA; 4 Department of Anesthesiology, Perioperative Care, and Pain Medicine, New York University, New York, USA; 5 Department of Anesthesiology, Duke University, Durham, USA

**Keywords:** anesthesia, difficult airway management, non-invasive positive pressure ventilation, obstructive sleep apnea, superno2va

## Abstract

The nasal positive pressure oxygenation device, SuperNO2VA™ (Vyaire Medical™), represents a significant advancement in airway management and perioperative care, particularly in patients at high risk for hypoxemia. This paper traces the historical development, underlying technology, and clinical adoption of the device, emphasizing its role in improving patient outcomes. By addressing gaps in traditional airway management and leveraging innovative design, the SuperNO2VA™​​​​​​​ device has become a useful tool in modern anesthesiology.

## Introduction and background

Introduction

Airway management is the cornerstone of anesthesiology, with innovations continuously shaping clinical practice. The SuperNO2VA™ (Vyaire Medical™) device, short for "Super Nasal Oxygenation and Ventilation Apparatus," was developed to address challenges in non-invasive ventilation, particularly during procedural sedation and in high-risk patient populations. This paper explores the historical context, development, and adoption of the SuperNO2VA™ device.

Historical context of airway management

Airway management has significantly progressed over time. Early techniques included optimal positioning to relieve airway obstruction, likely corresponding to what we now recognize as the head tilt-chin lift and jaw thrust maneuvers. This evolved into the Guedel and Berman oral airways in the 20th century, designed to keep the tongue clear of the pharynx [1]. Nasopharyngeal airways, or nasal trumpets, provided an alternative when oral access was limited, particularly in patients who were drowsy but arousable [2]. These innovations paved the way for modern tools such as laryngoscopes, supraglottic devices such as the laryngeal mask airway (LMA), video-assisted intubation systems, and non-invasive oxygenation devices such as high-flow nasal cannulas (HFNC) and nasal non-invasive positive pressure oxygenation devices. These advancements are summarized in Table 1. A comparison between HFNC and nasal non-invasive positive pressure oxygenation devices is made in Table 2.

**Table 1:** Summary of the history of advancements in airway management technology LMA: laryngeal mask airway, HFNC: high-flow nasal cannula Source: [1,2]

Era	Tool/technique	Description
18th-19th century	Oropharyngeal manipulation	Manual positioning to relieve airway obstruction
1930s	Guedel airway	Standardized oral airway to prevent tongue occlusion
Mid-20th century	Nasopharyngeal airway (nasal trumpet)	Flexible tubes for patients, bypassing oral access issues
1940s-1950s	Laryngoscope	Revolutionized intubation by providing a clear view of the glottis
1980s	LMA	Supraglottic airway device allowing for less invasive ventilation
2000s	Video-assisted laryngoscopes	Enabled video visualization during intubation
2010s	HFNC	Provide heated, humidified oxygen at high flow rates
2020s	Nasal non-invasive positive pressure oxygenation device	Maintains oxygenation and airway patency without invasive intubation

**Table 2:** Comparison between high-flow nasal cannula and nasal non-invasive positive pressure oxygenation device PEEP: positive end-expiratory pressure Source: [1]

Category	High-flow nasal cannula	Nasal non-invasive positive pressure device
Oxygen delivery	High-flow, humidified oxygen	Positive pressure oxygen via a sealed nasal mask
Primary use	Hypoxemic respiratory support, pre-oxygenation	Apneic oxygenation, procedural sedation, difficult airway
Positive pressure	Low-level PEEP (3-5 cm H_2_O)	Higher, adjustable positive pressure (up to 12 cm H_2_O)
Limitations	Less effective in apneic or obstructed airways	Requires a good seal; limited data in long-duration use

**Figure 1:**
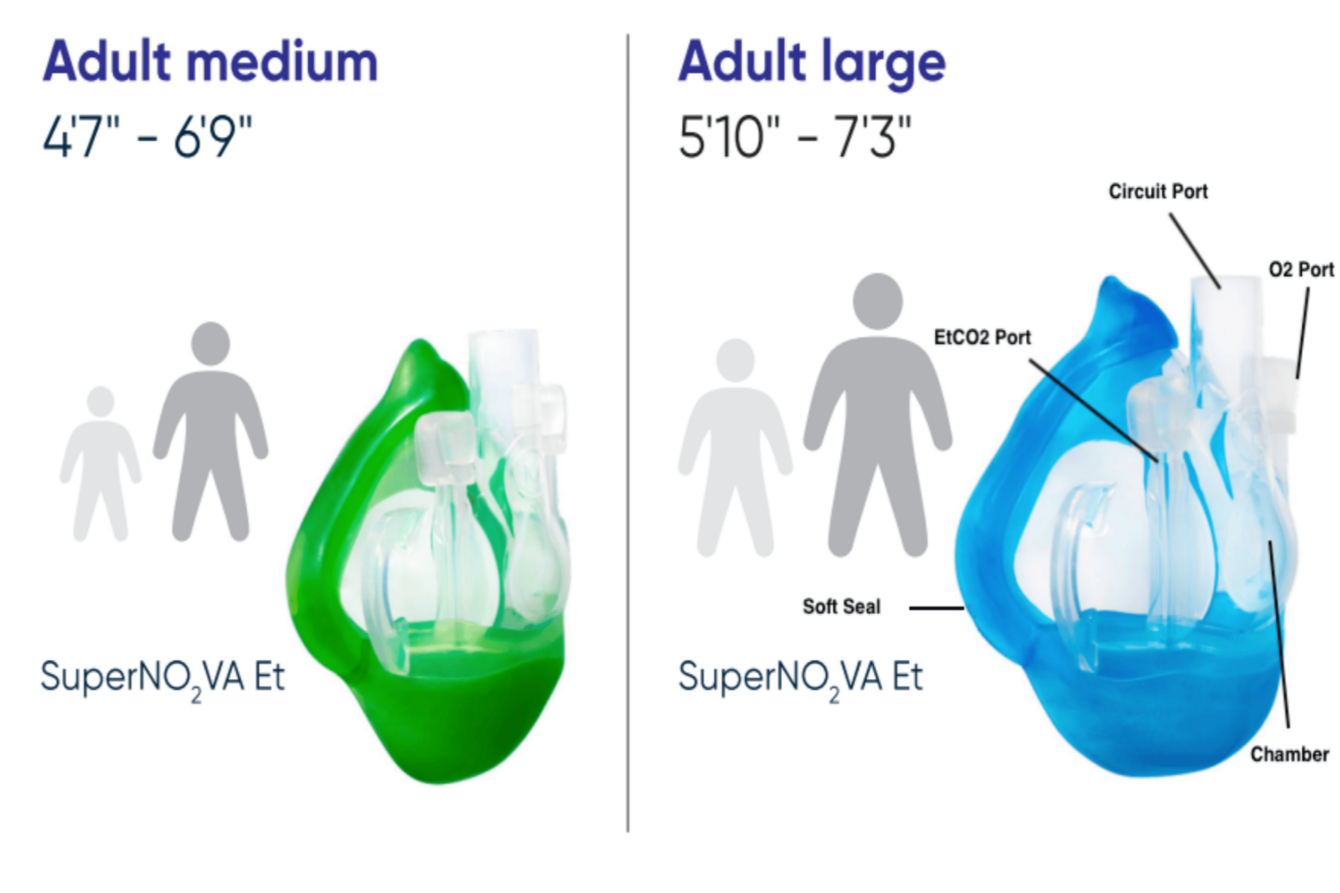
SuperNO2VA™, a nasal positive pressure oxygenation device used in modern airway management Permission to adapt and republish the original content was obtained from the original publisher. Attribution to the original source has been appropriately provided [4].

Despite advances in airway management, certain patient populations, such as those with obstructive sleep apnea (OSA) or morbid obesity, present unique challenges that necessitate alternative solutions. These challenges include increased neck circumference, larger tongue, and reduced neck flexion and extension [3]. The nasal positive airway pressure device, commonly known as SuperNO2VA™, emerged as a response to the needs unique to these populations with difficult airway management. This device, shown in Figure 1, is designed to prevent and relieve upper airway obstruction by delivering nasal positive pressure oxygenation, effectively stenting open the upper airway to maintain patency.

## Review

Development of the SuperNO2VA™ device

The SuperNO2VA™ device was developed by Vyaire Medical™ in 2016 with the goal of providing effective oxygenation and ventilation without the need for invasive techniques. The device's design integrates an anatomically shaped nasal mask with dual functionalities: continuous oxygenation and assisted ventilation. This dual capability allows clinicians to maintain airway patency while delivering positive pressure ventilation, effectively bridging the gap between traditional nasal cannula and more invasive devices.

Key milestones in its development include the following, which are also summarized in Table 3.

**Table 3:** Summary of the key milestones in the development of the nasal non-invasive positive pressure oxygenation device FDA: Food and Drug Administration Source: [4-6]

Year	Milestone	Details
2016	Design and introduction	The device was developed for nasal positive pressure ventilation in clinical settings [4].
2016	FDA regulatory approval	FDA clearance was received for short-term use of the device in adult patients in various clinical settings.
2020	Clinical protocol development	Vyaire Medical™ published a clinical use protocol for the device, detailing guidelines for operating room and anesthesia care.
2021	Assessment of efficiency and safety in endoscopic procedures	The device was evaluated for safety and efficiency in patients undergoing endoscopic procedures, showing positive results [5].

Design and Introduction (2016)

The device was developed by Vyaire Medical™ to provide nasal positive pressure ventilation, optimized for various clinical settings, including preoperative, intraoperative, and postoperative care. Its design aimed to address airway management challenges and reduce reliance on more complex ventilation systems [4].

FDA Regulatory Approval (2016)

The device received FDA clearance for short-term use in adult patients, affirming its safety for clinical use [6].

Clinical Protocol Development (2020)

Vyaire Medical™ published a clinical protocol for standardized use of the device in the operating room (OR), non-OR anesthesia areas, and post-anesthesia care unit (PACU). It outlines the setup by securing the device over the patient's nose, delivering oxygen at 10 liters per minute (LPM), and confirming positive pressure via respiratory bag inflation. When used with an anesthesia machine, the adjustable pressure-limiting (APL) valve is set to 10 cmH2O. Weaning involves reducing pressure support, monitoring for spontaneous breathing, and transitioning to low-flow oxygen once the patient is awake [4].

Assessment of Efficiency and Safety (2021)

A study assessed the safety and efficiency of the nasal non-invasive positive pressure oxygenation device in patients undergoing endoscopic procedures. The research concluded that the device effectively provided positive airway pressure, reducing dead space and enhancing patient safety during sedation [5].

The SuperNO2VA™ device is a non-invasive ventilation tool designed to optimize oxygenation and airway stability during various medical procedures [5]. By delivering continuous positive airway pressure through a nasal interface, it serves as an alternative to traditional preoxygenation and airway support methods. This device has gained attention for its ability to mitigate perioperative hypoxemia, particularly in patients with compromised respiratory function [5]. While its clinical utility spans multiple specialties, its integration into routine practice necessitates an understanding of both its advantages and limitations. The following section explores its clinical applications, highlighting its role in specific patient populations and addressing potential drawbacks.

Clinical applications and benefits

The SuperNO2VA™ device has demonstrated significant benefits in various clinical contexts, summarized in Table 4.

**Table 4:** Summary of the significant benefits and downsides of the nasal non-invasive positive pressure oxygenation device EtCO₂: end-tidal carbon dioxide Source: [7-9]

Category	Details
Benefits	
Obstructive sleep apnea	Non-invasive positive pressure support reduces hypoxemia risk during procedures [7]
Morbid obesity	Enhances oxygenation and ventilation in patients with reduced functional residual capacity
Difficult airway management	Pre-intubation oxygenation tool minimizes desaturation events
Limitations	
Cost	May not be cost-effective for all institutions
Operator learning curve	Proper training is essential; suboptimal use may reduce efficacy
Limited applicability	Unsuitable for patients with facial trauma, nasal obstruction, or other contraindications to non-invasive ventilation
Device dependence	May lead to reduced emphasis on alternative airway management techniques
Seal dependency	Requires a tight facial seal
Monitoring workaround	EtCO₂ requires an improvised setup
Outcomes	No proven mortality benefit

Benefits

Obstructive sleep apnea (OSA): The nasal non-invasive positive pressure oxygenation device provides non-invasive positive pressure during procedures, reducing the risk of hypoxemia, particularly in patients with OSA undergoing sedation or anesthesia [8].

Morbid obesity: It enhances oxygenation and ventilation in patients with reduced functional residual capacity, improving oxygen saturation during high-risk procedures such as bariatric surgery [8].

Difficult airway management: The device serves as a pre-intubation oxygenation tool, minimizing desaturation events, as demonstrated in a prospective study comparing patients undergoing difficult airway management with and without the device, which showed a significant reduction in desaturation events compared to traditional methods [7].

Clinical studies have demonstrated a marked reduction in perioperative complications, including hypoxemia, hypercapnia, and procedural interruptions due to airway instability [9].

Limitations

Cost: The device may not be cost-effective for all institutions, particularly those with limited budgets.

Operator learning curve: Effective use requires proper training, and suboptimal application, such as poor mask positioning or inadequate seal, may reduce its efficacy.

Seal dependency and fit challenges: A limitation of the device is its dependence on achieving a strong facial seal to deliver positive pressure. Many patients present with facial anatomy that makes it difficult to maintain this seal without applying excessive pressure, which can lead to discomfort or ineffectiveness.

Limited applicability: It may not be suitable for patients with significant facial trauma, nasal obstruction, or other contraindications to non-invasive ventilation.

**Figure 2:**
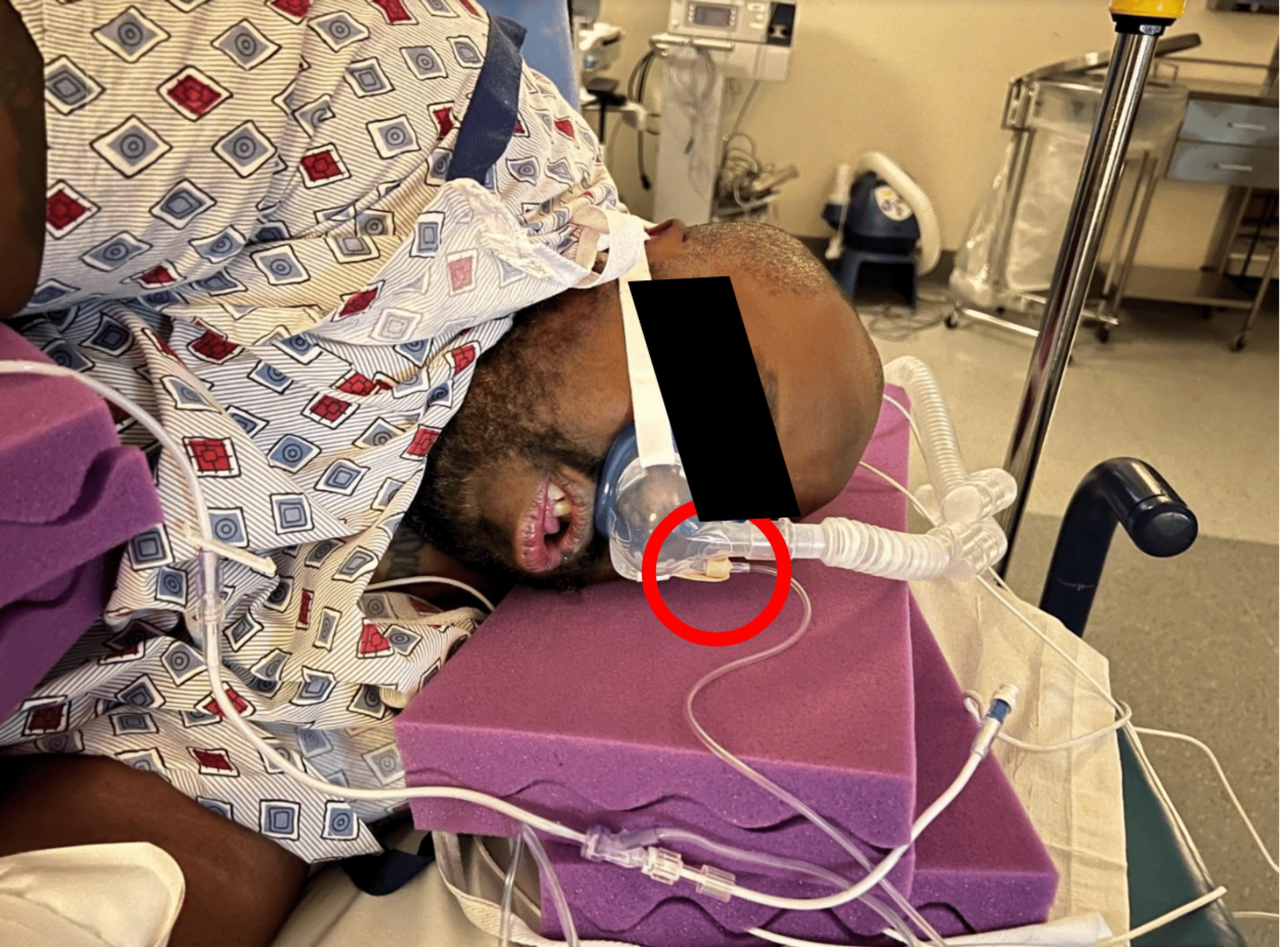
How clinicians often tape the sampling line to the oxygen port due to a lack of a luer lock, which may affect accuracy (red circle) Original image. Published with written informed consent from the patient.

Monitoring limitations: The device is not designed for end-tidal carbon dioxide (EtCO₂) monitoring; clinicians often tape the sampling line to the oxygen port due to a lack of a luer lock, which may affect accuracy. This is seen in Figure 2.

Outcomes: It reduces desaturations and interruptions [7], but no evidence yet of improved mortality or major morbidity. Benefits appear more related to efficiency and avoiding general anesthesia.

Device dependence: Reliance on the device may lead to a reduced emphasis on mastering alternative airway management techniques.

Adoption and impact on practice

Since its introduction, the SuperNO2VA™​​​​​​​ device has been integrated into diverse clinical settings, including operating rooms, endoscopy suites, and emergency departments. Educational initiatives and professional endorsements have further accelerated its adoption. The device has not only improved patient outcomes, such as maintaining adequate oxygenation during sedation, reducing hypoxic episodes, and preventing the need for escalation to endotracheal intubation, but also reduced the burden on healthcare systems by minimizing the need for more invasive and resource-intensive airway management techniques [9].

Future directions

Ongoing research aims to expand the applications of the nasal positive pressure ventilation devices in critical care and outpatient settings. Many clinical trials focus on the efficacy of positive pressure nasal ventilation as a means of respiratory support following extubation, particularly in neonate populations [10,11]. Innovations in material design and integration with monitoring technologies show promise for further enhancing its efficacy and utility. Compared to other oxygenation devices, such as transnasal humidified rapid-insufflation ventilatory exchange (THRIVE), nasal positive pressure devices maintain airway patency using a sealed mask, which may be especially useful in cases of airway obstruction [12]. However, further comparative studies are needed to evaluate the relative advantages of each approach.

## Conclusions

The nasal positive pressure oxygenation device demonstrates the transformative impact of targeted innovation in medical technology. By addressing long-standing challenges in airway management, it has significantly improved patient safety and procedural efficiency. Further randomized controlled trials are necessary to establish the device's applicability and feasibility in clinical anesthesia practice. Although no studies currently evaluate its utility, benefits, or limitations in difficult airway scenarios such as "cannot intubate, cannot oxygenate" (CICO), these critical situations warrant further investigation. As clinical evidence continues to support its benefits, the device's role in modern anesthesiology is likely to expand, making it part of the foundation of contemporary airway management strategies.
